# Could the breed composition improve performance and change the enteric methane emissions from beef cattle in a tropical intensive production system?

**DOI:** 10.1371/journal.pone.0220247

**Published:** 2019-07-26

**Authors:** Isabella Cristina de Faria Maciel, Fabiano Alvim Barbosa, Thierry Ribeiro Tomich, Luiz Gustavo Pereira Ribeiro, Ramon Costa Alvarenga, Leandro Sâmia Lopes, Victor Marco Rocha Malacco, Jason E. Rowntree, Logan R. Thompson, Ângela Maria Quintão Lana

**Affiliations:** 1 Federal University of Minas Gerais, Department of Animal Science, Belo Horizonte, MG, Brazil; 2 Michigan State University, Department of Animal Science, East Lansing, MI, United States of America; 3 De Heus Animal Nutrition B.V., Rubensstraat VE Ede, The Netherlands; 4 Embrapa Dairy Cattle, Juiz de Fora, MG, Brazil; 5 Embrapa Maize & Sorghum, Sete Lagoas, MG, Brazil; United States Department of Agriculture, Agricultural Research Service, UNITED STATES

## Abstract

Crossbreeding has been used to improve performance in beef cattle, however the effects of breed composition on methane (CH_4_) production, yield and intensity from cattle raised in tropical intensive and integrated systems remain unknown. To assess the impact of breed composition on performance and methane emissions, Nellore (NEL; yr 1: BW = 171.5 ± 19.4 kg; n = 10; yr 2: BW = 215.8 ± 32.3 kg, n = 25) and Angus x Nellore crossbred (AN; yr 1: BW = 214.2 ± 26.4 kg, n = 10; yr 2: BW = 242.5 ± 32.2 kg, n = 25) were compared. The animals grazed on integrated crop-livestock system in the growing phase (stocking rate 2452 kg BW/ha, herbage mass 4,884 kg dry matter (DM)/ha, forage allowance 5.9 kg DM/100kg BW) and then were finished in a feedlot. Steers (n = 8) from each breed composition were randomly selected in each phase to measure CH_4_ production using a sulfur hexafluoride (SF_6_) tracer technique and DM intake (DMI) using titanium dioxide. Compared with NEL, AN had both superior total gain and average daily gain (ADG) in the grazing period. The AN presented greater ADG in the feedlot with a shorter finishing period and resulted in greater carcass yield and carcass ADG. Methane production (kg/period) was lower in NEL (19% less) than AN in grazing (*P*<0.01), and no difference was observed in feedlot. The NEL had less CH_4_ intensity (CH_4_/BW) in grazing but greater CH_4_ per unit of ADG in the feedlot compared to AN. Breed composition did not influence the CH_4_ yield (CH_4_/DMI) in either phase, despite the difference in feedlot DMI (kg/day). In conclusion, crossbreeding may be an option to improve performance and reduce the CH_4_ per ADG in tropical climate conditions, resulting in lower methane emission per kg of meat produced.

## Introduction

The population around the world has been growing rapidly and has a corresponding increase in food demand. The improvement in environmental efficiency of beef production systems seems to be, at least for the foreseeable future, part of the solution for the issue of global food security [1]. Notwithstanding, ruminant livestock systems are under continued political pressure to reduce their greenhouse gas (GHG) outputs.

Cattle production is an important driver for Brazil’s economy, and ranks second worldwide, with approximately 212 million head [2]. Additionally, Brazil is the largest beef exporter, maintaining trade relations with 180 countries. Traditionally, the national herd is maintained in an extensive pasture-based production system. However, more recently, there has been a notable shift in Brazilian beef production, with livestock farming gradually occupying less land with increased production and productivity gains [3].

The modern, intensive livestock systems, like beef production in grain-finishing systems, offer both substantially lower land requirements and greenhouse gases (GHG) emissions per kilogram of meat than traditional, extensive ones [4]. However, the GHG emissions by ruminants in adaptive grazing systems has been shown in some studies [5, 6], to be considerably lower than previously thought. This decrease was attributed to the quality and productivity of the pastures, potentially increasing soil carbon sequestration thereby negating atmospheric emissions [7]. Therefore, the best option could be a system that incorporates well-managed grass systems complemented by grain-fed components as cattle reach compositional maturity.

On the other hand, genetic improvement in beef cattle has a potential for reducing CH_4_ emissions [8, 9]. The Zebu (*Bos indicus*) animals, for example, are quite resistant and adaptable to tropical climates and, because of that, the Nellore is the most prevalent breed in Brazil. However, *Bos taurus* animals demonstrate greater yield potential, especially under appropriate conditions [10]. Thus, crossing breeds could be a viable alternative to improve the production rates of purebred cattle in tropical climates. Faster-growing animals can be more efficient in quantity of product produced, because they should theoretically partition relatively more feed nutrients into production. Thus the output of polluting excretion products on a per unit product basis should be less for these animals [11].

Due to the contribution of livestock in GHG, there is a strong motivation for the measurement of enteric CH_4_ to be accurately performed. Besides this, methane emission inventories are based on models developed in temperate climates and, therefore, precise methane measurements of tropical region production systems are crucial to reduce the uncertainties of these inventories and evaluate GHG mitigation strategies [12].

The objective of this trial was to examine the animal performance and enteric CH_4_ production, yield and intensity from two breed compositions in a Brazilian beef cattle production system–rearing in an integrated crop-livestock system but finished in a feedlot.

Our hypothesis was that: *(i)* Performance of crossbred animals would be superior than Nellore in a Brazilian beef cattle production system; and (*ii*) CH_4_ yield and intensity would be lower for crossbred animals compared to Nellore.

## Materials and methods

### Treatments and experimental design

The experiment was conducted at Brazilian Agricultural Research Corporation–Embrapa Maize & Sorghum (Sete Lagoas, Minas Gerais, Brazil; 19°28′S; 44°15′W, at 732 m altitude). Climate data for the experimental period was obtained at the meteorological station located at Embrapa and are presented in S1 Fig.

All experimental procedures used in this experiment were approved by the Ethics Committee for Animal Use of Universidade Federal de Minas Gerais (UFMG, protocol number 326/2014).

At trial onset, 10 mo old steers were divided into two groups according to their breed composition as follows: Nellore (171.5 ± 19.47 kg, n = 10), Angus x Nellore crossbred (214.2 ± 26.41 kg, n = 10) in the first year and Nellore (215.8 ± 32.34 kg, n = 25), Angus x Nellore crossbred (242.5 ± 32.26 kg, n = 25) in Year 2.

### Grazing management

The animals were evaluated in the rearing period, with initial age of 10 months, in the integrated crop-livestock (ICL) system under no-tillage system adopted since 2005.

The pasture consisted of *Megathyrsus maximus* cv. Mombaça and the total pasture area of 5.5 hectares (ha) was subdivided into five sub-paddocks of approximately 1.1 ha each, used as a rotational grazing system with seven days grazing period and 28 days of rest. After each grazing cycle, the paddocks were fertilized with 150 kg of Nitrogen/ha. The experimental grazing period lasted 230 and 216 days in the first and second year, respectively. All animals were drenched with an anthelmintic prior to the start of grazing.

The energetic-protein supplement (Table 1) was offered *ad libitum* throughout the grazing period in a collective feeder. Supplement daily intake was estimated by dividing the total supplement consumed by the number of animals for each day in each period. As the supplement was offered *ad libitum* in a collective feeder, consumption might be different among animals related to self-intake regulations.

**Table 1:** Percentage of ingredients of the energy-protein mineral supplement used in pasture test and TMR diet used in feedlot.

Ingredients (%DM)	Pasture Supplement		Feedlot TMR diet
	Year 1	Year 2	
**Corn Silage**	-	-	35
**Corn grain ground**	-	-	54
**Corn gluten meal**	84	86	-
**Soybean meal**	5	7	5
**Mineral Salt**^**a**^	11	7	6

^a^Amounts of minerals (per kg of supplement): Year 1: phosphorus (P), 9 g; calcium (Ca), 20 g; sulfur (S), 16 g; magnesium (Mg), 2 g; sodium (Na), 37 g; zinc (Zn), 600 mg; copper (Cu), 150 mg; manganese (Mn), 140 mg; cobalt (Co), 20 mg; iodine (I), 17 mg; selenium (Se), 3 mg; iron (Fe), 100 mg.

Year 2: P, 6 g; Ca, 20 g; S, 16 g; Mg, 1.4 g; Na, 9 g; Zn, 450 mg; Cu, 100 mg; Mn, 100 mg; Co, 14 mg; I, 12 mg; Se, 2 mg; Fe, 100 mg. Feedlot: P, 18 g; Ca, 50 g; S, 10 g; Mg, 20 g; Na, 30 g; Zn, 1303 mg; Cu, 375 mg; Fe, 500 mg; Mn, 520 mg; Co, 50 mg; I, 50 mg; Se, 9 mg; Fe, 500 mg; lasalocid sodium, 450 mg.

Available herbage mass (AHM) was sampled within each paddock by cutting 5 randomly selected quadrats (1.0 m × 1.0 m) to ground level (5-cm stubble height) using hand shears before grazing. All collected herbage from each strip was collected, weighed and subsampled. A subsample (fresh weight) of the herbage sample from each quadrats was dried for 72 h at 65°C and was taken for subsequent chemical analysis.

A further subsample was manually separated in leaf, stem, and dead content, and was dried for 72 h at 65°C. Leaves were used to characterize the composition of the forage ingested by the animals. It was decided to evaluate only leaf, since it represented almost all the forage sampled (above 60%).

The forage allowance (kg dry matter [DM]/100 kg BW/day) was calculated by the ratio of forage production (kg DM/day) to total body weight of animals. In year one, there were 20 additional testers animals, that did not belong to the evaluated genetic groups and remained on pasture throughout all the experimental period.

### Feedlot management

In the feedlot, the animals were divided into groups according to the breed composition. The feedlot period began in June of each year, and the animals were allocated to collective pens measuring 20 x 12 m each and equipped with feed lanes and drinkers. The pens had enough space to ensure adequate animal well-being, with the minimal 18.5 m^2^ area per animal, observed in pens with 13 animals (year 2). All animals were drenched with an anthelmintic prior to the start of feedlot.

The cattle were fed three times per day–at 0700, 1100 and 1600 h. The ration was adjusted daily to maintain 5 to 10% refusals. The amount of feed offered was recorded per pen, and refusals were weighed daily. Feed samples were taken monthly for chemical analysis.

The animals were adapted to the experimental diets for 21 days. Initially, 60% corn silage and 40% concentrate diet were supplied, the amount of concentrate was increased until the ratio of roughage: concentrate was 35:65 (DM base). The diet was formulated to allow for 1.4 kg average daily weight gain [13] and consisted of corn silage, ground corn, soybean meal, and trace mineral mixture (Table 1).

A gain of 200 kg BW during the feedlot period was stipulated as the slaughter criterion. Animals remained in feedlot for 111 and 105 days (AN) and 138 and 127 days (NEL) in the first and second year, respectively.

Animal performance was determined monthly by recording body weight (BW) following a fast of feed and water for 16 hours. The average daily gain (ADG) was calculated as the difference between the final body weight (FBW) and the initial body weight (IBW) of each period (grazing and feedlot), divided by the total number of days.

On the day of slaughter, animals were weighed in the morning, before being sent to the slaughterhouse, where they were kept fasting for 24 hours with only *ad libitum* water intake. All the animals were slaughtered in a commercial slaughterhouse, according to the humanitarian procedures required by Brazilian legislation. The weight of hot carcass (WHC) was recorded immediately after the carcass was cleaned. Carcass yield (CY) was calculated by the ratio of WHC to FBW. The mean daily weight gain of carcass (ADGc) was calculated according to Eq (1):

$$ADGc=\frac{\left[WHC-(IBW\times 50\%)\right]}{days\;in\;feedlot}$$ (1)

### Methane production measurement

Enteric CH_4_ emissions were measured using the sulfur hexafluoride (SF_6_) tracer technique reported by [14] and modified by [15] during three periods—feedlot in first year, grazing and feedlot in second year. Technical problems prevented the measurement of methane in the first year of grazing.

Eight animals from both breed composition were evaluated in each period. Enteric CH_4_ emissions were measured for at least 3 days per animal. According to [16] a 3-days period is necessary to achieve an R of 0.70 for CH_4_ emissions by SF_6_ technique and the number of required animals to detect a difference of 20% in CH_4_ emissions among treatments is 6–8 animals per group.

Ten days before the beginning of each measurement, a SF_6_ permeation tube was introduced directly into the rumen of each animal via the esophagus. The permeation rates were 4.44 ± 0.28; 4.60 ± 0.39 and 4.29 ± 0.06 mg/d (mean ± SD) in feedlot first year, grazing second year, and feedlot second year, respectively, as given by an 8-weeks calibration assay in a controlled environment at 39°C.

Expired gases were collected with a sampling apparatus containing a collection canister made of polyvinyl chloride (PVC) equipped with a capillary tube (0.127 mm diameter). The capillary was calibrated to allow the vacuum inside the canister remaining at 40–60% of the initial vacuum after 24 h of measurement. If the pressure inside the canisters was below or above the 40–60% range, gas samples were not collected. Additionally, an identical set was used to collect background air samples at two points at the same time canisters were collected from animals.

Canisters were removed daily at 0900 h, evacuated, and replaced then the contents were sampled. Animals were moved to a chute area for each canister evacuation, and total time to sample and replace canisters for all animals in both breed compositions groups was approximately 1 h. To collect enteric CH_4_ and SF_6_ samples, the canisters were vacuumed to approximately −12 PSI with vacuum pump. After the collection period, canisters were individually connected to a dilution system, and the final pressure was recorded. Nitrogen was then added slowly until canister pressure reached +13 PSI. Pressure readings were recorded to calculate the dilution factor [17]. After pressurization, the contents of the canisters were transferred under positive pressure to four pre-evacuated 20 mL Exetainers vials (Labco Limited, Lampeter, UK) for each animal.

The collected respired air were analyzed immediately after the end of the experimental period. Analysis of CH_4_ and SF_6_ concentrations were determined by gas chromatography at the Laboratory of Gas Chromatography, Embrapa Dairy Cattle, in Juiz de Fora, Minas Gerais, Brazil. The SF_6_ (ppt) and CH_4_ (ppm) concentrations in the sampling canisters were determined using two separate gas chromatographs; models 6890 N plus and 7820A, respectively (Agilent Technologies, Santa Clara, CA). Both chromatographs were equipped with a split-splitless injector, but a μECD detector (electron capture) was used to measure SF_6_ and a FID detector (flame ionization) was used to measure CH_4_ concentration.

For SF_6_ analysis, a column (HP-Molsieve, Agilent Technologies, Santa Clara, CA) was used with N_2_ as carrier gas at a flow rate of 5.0 mL/min with N_2_ as the makeup gas at 40 mL/min, with μECD detector. The gas chromatograph was calibrated weekly using SF_6_ (White Martins, São Cristóvão, RJ) standards ranging in concentrations from 30, 100, 500, 1500, 3000 ppt. The CH_4_ was analyzed using two columns, (HP-Plot/Q and HP-Molsieve, Agilent Technologies, Santa Clara, CA) with H_2_ as carrier gas at a flow rate of 7.0 mL/min, with FID detector. The gas chromatograph was calibrated using CH_4_ (Linde AG, Rio de Janeiro, RJ) at 4.8, 9.7, 19.6, 102, 203 ppm.

The CH_4_ emission rate (RCH_4_, g/d) for each animal was calculated using the SF_6_ and CH_4_ mixing ratio (μmol/mol) sampled by the canisters on the animals (SF_6_ and CH_4_ canister, respectively) and those used for background (SF_6_ and CH_4_ background, respectively), and the predetermined SF_6_ release rate (RSF_6_, g/d) from the permeation tubes, where molecular weights (MW) of the gases is MWCH_4_ = 16 and MWSF_6_ = 146, as described by [18], using Eq (2):

$${RCH}_{4}={RSF}_{6}\times \left[\frac{\left({CH}_{4}canister-{CH}_{4}background\right)}{\left({SF}_{6}canister-{CH}_{6}background\right)}\right]\times \left[\frac{{MWCH}_{4}}{{MWSF}_{6}}\right]\times 100$$ (2)

Individual animal methane emissions were expressed as methane production (g CH_4_/animal/day, kg CH_4_/year, and kg CH_4_/period), methane yield (g CH_4_/kg DMI) and methane intensity (g CH_4_/kg BW), besides g CH_4_/kg ADG.

### Intake measurement

Individual DMI was determined for eight animals from each group in each period (grazing or feedlot, year 1 and 2), the same animals used for the methane measurement. Titanium dioxide (TiO_2_) was used as intake marker, and 10 g were administered to the animals once daily for 12 days during each period. TiO_2_ was stored in paper cartridges and introduced directly into the esophagus of the animals at 0900 h with the aid of a PVC applicator.

Fecal samples were collected once daily during the last 5 days of the dosage period. Samples of feces corresponding to the different collection times composed a sample for each animal. Feces were dried at 65°C until constant weight. Dried feces were ground through a 1mm screen with a Wiley mill and analyzed by atomic absorption spectrophotometry.

The TiO_2_ content was determined according to [19]. The standard curve was prepared using 2, 4, 6, 8 and 10 mg TiO_2_ and the spectrophotometer readings were recorded at a wavelength of 410 nm. For the calculation of fecal production (FP) estimated by TiO_2_, the following formula was used:

$$FP=\frac{{TiO}_{2}supplied}{{TiO}_{2}in\;feces/DM\;105{}^{\circ}C}$$ (3)

where FP = fecal production obtained by TiO_2_, g DM/day; TiO_2_ supplied = amount of TiO_2_ supplied to the animals per day (10 g); TiO_2_ in feces = percentage of titanium in feces, %; DM 105°C = the dry matter of feces at 105°C.

Fecal Production and indigestible NDF (iNDF) were used to estimate dry matter intake (DMI, kg/day) for each animal. Indigestible NDF was used as the internal marker and obtained after *in situ* incubation of a diet (iNDF diet) and feces (iNDF feces) samples for 288 hours in the rumen of a cannulated bovine [13]. Follow equation (Eq 4) was used for DMI:

$$DMI=FP\times \left(\frac{iNDF\;feces}{iNDF\;diet}\right)$$ (4)

Average daily DMI during the methane measurement period and CH_4_ emission rate were used to calculate methane yield (g CH_4_/kg DMI).

The average BW, ADG, DMI and feed and conversion efficiency were calculated over the same CH_4_ measurement period in both grazing and feedlot.

### Chemical analysis

Forage samples, supplements, diets, and refusals of foods were collected, oven-dried in a forced-ventilation oven at 65°C, for at least 72 hours, and ground in a Wiley mill (Alpax, Diadema, SP, Brazil) through a 1-mm sieve.

The constituents were determined as described by [20], according to the following methods: dry matter (DM), 934.01; crude protein (CP), 984.13 (Leco FP-428, Australia Pty Ltd., Castle Hill, New South Wales, Australia); neutral detergent fiber (NDF), 2002.04; acid detergent fiber (ADF), 973.18; ether extract, 920.85; and ash (500°C furnace for 6 h), 938.08.

### Statistical analysis

To evaluate animal performance, a completely randomized design was used. Data for daily DMI were averaged per animal per 5-d period. The methane production data was averaged per animal per 3-d period minimum.

Breed composition, year and the interaction between year and breed were included in the model, as a fixed effect. The distribution of model residuals was tested for normality using Shapiro-Wilk W test and for uniformity using the Cochran test.

The mathematical model used was: Y_ijk_ = μ + B_i_ + Y_j_ (BY)_ij_ + ε_ijk_, in which: Y_ij_ is the observation of the animal k, from the breed i, in year j, μ is the mean effect; B_i_ is the fixed effect of the breed composition i, (i = 1, 2); Y_j_ is the fixed effect of the year j, (j = 1, 2); (BY)_ij_ is the interaction effect breed i and year j and ε_ijk_ is the random error associated with each animal.

Statistical analysis was performed using PROC GLM from SAS software (version 9.2; SAS Inst. Inc., Cary, NC). Means were compared using the Fisher’s test. Treatment differences were considered significant at *P*<0.05.

## Results and discussion

### Grazing and feedlot diet characteristics

Forage production during the grazing period was satisfactory and corresponded with average herbage mass (AHM) of approximately 3,884 kg DM/ha. Stocking rate was higher in the second year (2880 versus 2025 kg BW/ha in the first year), and forage allowance (kg DM/100 kg BW) was 6.9 and 4.9 in the first and second year, respectively (Table 2).

**Table 2:** Forage characteristics and productivity for grazing and feedlot system for each year in an intensive beef cattle production system.

System	Item	Year 1	Year 2
**Grazing**	N° animals	40	50
	Days in grazing	230	216
	Herbage Mass, kg DM/ha	3,824	3,944
	Stocking Rate, kg BW/ha	2025	2880
	Forage Allowance, kg DM/100 kg BW	6.9	4.9
	Total Gain, kg/animal	166.7	156.3
	Total Gain, kg BW	6660	7800
**Feedlot**	Days in feedlot	125	116
	Total Gain, kg/animal	175.8	189.2
	Total Gain, kg BW	7020	9450

DM, dry matter; BW, body weight.

Forage production and quality were not limiting allowing animals to achieve high gains during the grazing period (Tables 2 and 3). Forage analysis was performed on the leaves only. Leaves are preferentially grazed by the cattle when the availability of forage was not limiting [21]. We assumed that the animals had the opportunity to select and eat high quality plant material with nutritional composition more similar to that found in the leaves which justifies the use of this type of forage sampling for analysis.

**Table 3:** Chemical composition of *Megathyrsus maximus* 'Mombaça' pasture, of the supplement and of the TMR diet offered in the feedlot for the two breed compositions during experimental period.

Item	Grazing Period^a^				Feedlot Period			
	Year 1		Year 2		Year 1		Year 2	
	Forage	Supplement	Forage	Supplement	NEL	AN	NEL	AN
**DM, %**	25.49	86.78	27.8	90.29	59.94	60.09	57.94	58.61
**Ash**^**b**^	8.06	26.27	7.24	23.95	3.60	3.50	4.23	4.34
**OM**^**b**^	88.44	65.77	86.04	72.75	92.28	92.46	86.81	86.75
**CP**^**b**^	12.7	20.68	13.24	20.87	15.31	15.52	16.03	16.02
**EE**^**b**^	1.78	4.09	2.05	3.41	3.75	3.71	4.09	4.28
**NDF**^**b**^	64.34	27.23	67.01	28.74	26.40	25.97	27.48	27.30
**ADF**^**b**^	44.79	8.40	35.51	8.02	12.19	12.06	11.81	11.65
**Hem**^**b**^	34.48	18.83	35.56	20.72	14.21	13.99	15.47	15.66
**Cel**^**b**^	41.36	7.41	32.54	7.13	10.39	10.32	11.22	11.13
**Lignin**^**b**^	3.43	0.99	2.97	0.89	1.80	1.74	0.59	0.52
**CC**^**b**^	28.30	72.76	28.91	71.26	73.60	75.55	72.51	72.69
**P**^**b**^	0.22	0.88	0.20	0.84	0.33	0.33	0.37	0.37
**Ca**^**b**^	0.64	4.18	0.69	3.43	0.39	0.40	0.55	0.53
**TDN, %**	56.95	70.00	55.84	74.00	75.93	76.18	75.31	75.42

^a^The grazing period was 1^st^ year– 10/29/2015 to 06/15/2016 and 2^nd^ year– 11/16/2016 to 06/20/2017.

^b^%DM; DM, dry matter; OM, Organic matter; CP, Crude protein; EE, Ethereal extract; NDF, Neutral detergent fiber; ADF, Acid detergent fiber; Hem, Hemicellulose; Cel, Celulose; CC, Cell content; P, Phosphorous; Ca, Calcium; TDN, Total digestible nutrients. The TDN was estimated using the formula recommended by [22]: TDN (%) = 83.790–4171 x NDF (forage) and TDN (%) = 91.0246 − 0.571588 x NDF (FL diet); NEL: Nellore; AN: Angus x Nellore crossbred.

The high herbage availability and CP during the experimental period may have resulted from nitrogen fertilization and from the use of the ICL system. As the ICL system has been improved over the years, the stocking rate was higher than that obtained in previous study executed in the same area during 2013/2014 [23]. The stocking rate was 1093.5 and 1431.0 kg BW/ha in the dry and rainy seasons, respectively [23], which was lower than the present study during the rainy period (2880 and 2025 0 kg BW/ha in the first and second year, respectively). This difference was attributed to the greater number of animals used in the current study.

The greater stocking rate maintained by our grazing strategy led to a more efficient forage utilization resulting in more and higher quality forage throughout the grazing season. [24] simulated scenarios for beef production in Brazil and indicated the best scenario was similar to the system in this study (Nellore and Nellore crosses animals in rearing phase in rotational grazing), and resulted in a lower stocking rate (1237.5 kg BW/ha), which attests the potential of ICL systems to increase the animals’ production.

### Animal performance

There was an effect of the breed composition on the performance variables in both the grazing and feedlot period (Table 4). Due to the inherent properties of hybrid vigor, the AN sired cattle began the trial with a greater BW in relation to NEL, despite them being comparably aged.

**Table 4:** Effects of breed composition on animal performance of beef cattle in grazing and feedlot tests (where NEL = Nellore, AN = Angus x Nellore crossbred).

	NEL (n = 35)	AN (n = 35)	SEM	P Value		
				Breed	Year	Breed *Year
**Grazing**						
Initial Weight, kg	203.13	234.44	5.55	<0.01	<0.01	0.28
Final Weight, kg	351.71	404.41	7.94	<0.01	<0.05	0.15
Total Gain, kg	148.58	169.97	4.19	<0.01	0.09	0.14
ADG, kg/animal/d	0.675	0.772	0.01	<0.01	>0.10	0.19
**Feedlot**						
Initial Weight, kg	337.74	418.38	6.40	<0.01	<0.01	>0.10
Final Weight, kg	509.41	617.45	9.72	<0.01	<0.01	0.16
Total Gain, kg	171.67	199.07	4.88	<0.01	<0.05	<0.05
ADG, kg/d	1.320	1.869	0.04	<0.01	<0.01	<0.05
Carcass Weight, kg	284.23	352.43	5.79	<0.01	<0.05	0.10
Carcass Yield, %	55.79	57.08	0.27	<0.01	<0.01	0.18
Carcass ADG FL, kg/d	0.886	1.344	0.03	<0.01	<0.01	<0.05
Carcass ADG Total, kg/d	0.521	0.721	0.01	<0.01	0.06	<0.05

ADG, average daily gain; FL, feedlot; SEM, standard error of the mean.

Total gain and ADG in the grazing period were higher for AN (*P*<0.01) and, consequently, they presented greater weight at the end of this period (*P*<0.01) (Table 4). The weight gains obtained in the current study were higher than those reported by [25] with similar levels of DMI. These authors evaluated Nellore animals (initial weight of 373 kg) in continuous grazing put-and-take stocking of *Urochloa brizantha* Stapf cv. Marandu and the animals obtained DMI of 5.93 kg/day and ADG of 0.447 kg/day, compared to 0.675 and 0.772 kg/d for Nel and AN cattle in the current study.

Voluntary intake of forage was estimated by use of external and internal markers. The estimation of feed intake in pasture-raised animals continues to be costly and highly variable, despite advances in the experimental and analytical procedures over time [25]. However, in this study, the DMI values obtained for grazing animals are in accordance with [26] (5.90 kg DMI/d for NEL vs. 6.23 kg DMI/d for AN).

These results show the capacity for improved animal production per area in ICL systems. Although the beef cattle sector in Brazil is still characterized by regions with low efficiency indexes [27], ICL systems could improve animal production and reduce environmental impacts from livestock in pasture-based beef production systems in the tropical regions.

In the feedlot period, there was a significant difference between the two breed compositions for all variables evaluated. The AN animals had higher ADG and feed conversion than NEL but did enter the feedlot at a significantly higher weight. The AN animals reached the desired finishing weight in 111 and 105 d in the first and second year, respectively. The NEL animals, although they remained in the feedlot longer (138 and 127 days in feedlot in first and second year, respectively) had lower total weight gain (172 kg) compared to AN.

Higher carcass weight were observed in AN animals when compared to NEL. The differences observed for carcass weight in this current study were related to differences in slaughter weight of the animals. Average finishing weights in the feedlot were similar to those reported in previous experiments using Angus cross and Nellore cattle [28, 29].

Breed composition had significant effect on carcass yield and carcass ADG (*P*<0.01), with AN animals being greater than NEL. Carcass ADG in feedlot was 35% higher for AN than NEL, while carcass ADG total (considered throughout the experiment period) was 28% higher for AN. This observed increase in productivity results in fewer finished animals needed to produce a given quantity of meat [30], which may contribute to reducing the environmental impacts of beef production.

Crossbred animals showed greater performance throughout the experimental period (total gain of 383 kg versus 306 kg for Nellore animals), but the growth rates reached by both breeds were satisfactory. High gains can be explained by the animals’ physiological conditions (non-castrated) and age (up to 24 months old) [31, 32], beyond the effect of cross breeding animals alone [33, 34], in addition to the high concentrate diet in the finishing phase.

Animal performance is not only a direct effect of the quality and quantity of the diet but also animal genetic potential [35, 36]. We observed that in appropriate conditions of feeding, AN animals obtain greater performance.

### Methane emissions

The effects of breed composition on methane emissions are presented in Table 5. It is important to note that due to technical issues with the methane measurement, methane emission measurements during the grazing period were only performed in year 2. Despite this, the focus of our study is not the comparison between years and the design of the study and the statistical analysis allowed us to discuss these data without leading us to partially misleading conclusions.

**Table 5:** Effects of breed composition on methane emissions of beef cattle in grazing and feedlot tests (where NEL = Nellore, AN = Angus x Nellore crossbred).

	NEL (n = 8)	AN (n = 8)	SEM	P Value		
				Breed	Year	Breed*Year
**Grazing**						
Total DMI, kg/day	5.90	6.23	0.31	0.66	-	-
Forage DMI	4.78	5.11	1.44	0.66	-	-
Supplement DMI	1.12	1.12	-	-	-	-
BW average, kg	314.6	336.6	9.33	0.07	-	-
ADG, kg/day	0.680	0.729	0.03	0.22	-	-
Feed Conversion	8.98	8.81	0.50	>0.10	-	-
Feed Efficiency	0.119	0.122	0.007	>0.10	-	-
CH_4_, g/day	79.69	98.05	4.45	<0.01	-	-
CH_4_, kg/year	29.08	35.78	1.62	<0.01	-	-
CH_4_, kg/period	17.21	21.17	0.85	<0.01	-	-
CH_4_, g/kg DMI	14.31	16.76	1.32	0.17	-	-
CH_4_, g/kg BW	0.24	0.28	0.05	<0.01	-	-
CH_4_, g/kg ADG	119.53	140.03	8.09	0.07	-	-
**Feedlot**						
Total DMI, kg/day	9.29	12.44	0.39	<0.01	0.10	<0.01
BW average, kg	386.2	488.6	4.87	<0.01	<0.01	0.25
ADG kg/day	1.49	2.26	0.07	<0.01	0.13	<0.05
Feed Conversion	7.17	5.93	0.36	0.06	0.05	<0.01
Feed Efficiency	0.167	0.193	0.009	0.09	>0.10	<0.01
CH_4_, g/day	168.72	209.84	7.78	<0.01	<0.01	>0.10
CH_4_, kg/year	61.58	76.59	2.84	<0.01	<0.01	>0.10
CH_4_, kg/period	22.34	22.67	0.98	>0.10	0.05	>0.10
CH_4_, g/kg DMI	18.52	17.83	0.89	>0.10	<0.05	<0.05
CH_4_, g/kg BW	0.43	0.42	0.11	0.73	0.08	0.66
CH_4_, g/kg ADG	122.76	97.49	6.86	<0.01	>0.10	0.06
CH_4_, g/kg CW	0.079	0.067	0.10	<0.01	0.16	0.52
CH_4_, g/kg ADGc	192.34	174.54	7.67	<0.05	<0.05	0.28

DMI, dry matter intake; BW, body weight; ADG, average daily gain; CH_4_, methane; CW, carcass weight; ADGc, ADG of carcass; SEM, standard error of the mean.

The CH_4_ production (g/day and kg/year) were lower in NEL than AN animals in both grazing and feedlot systems (*P*<0.01). Methane production emitted per period was calculated, according to grazing and feedlot days. The NEL emitted 19% less CH_4_ than AN in grazing, but no differences between breed composition in feedlot were observed (Table 5). Even though AN animals showed higher methane emission (g/day), the total methane emission during the finishing phase was the same for both breed compositions, because these animals spent less time in feedlot. The intensification of beef cattle production systems leads to a reduction in emissions of GHGs per unit of product, and greater reductions may theoretically be possible if animals of higher performance were utilized [24], as confirmed in this study by the Angus x Nellore cattle.

Methane production (g/day and kg/year) measured in grazing period were lower than those reported by [25]. The higher methane emission reported by these authors compared to the values obtained in this study may be attributed to the continuous grazing system used, where forage presents greater fiber content and therefore provides higher production of CH_4_. Compared to continuous grazing systems, multi paddock (MP) grazing can improve forage quality as well as forage production; thus, MP grazing is potentially a good option to reduce GHG emission [6]. According to [6], total GHG emissions could be reduced by as much as 30%, only by increasing forage quality and digestibility.

Although differences were observed for methane production (g/day and/or kg/year) measured in feedlot between the breed composition, the mean of methane emission were similar to those reported by other studies [37–39]. Feedlot diets generally do not exhibit many discrepancies in nutritional composition, and therefore lower methane emissions variations are observed at that stage.

It was found that there was no difference in DMI between breed compositions in pasture, but in the feedlot AN presented higher DMI than NEL (*P*<0.01). Despite the difference in DMI, breed composition did not influence the CH_4_ yield (g CH_4_ per unit of DMI) neither in pasture nor in feedlot. The AN consumed more feed in feedlot, however when CH_4_ volumes were compensated for feed intake, there were no significant differences between breeds. Methane production expressed as g/kg DMI in the current study was similar to previously observed production rates in Nellore animals by [37] (17.1 g de CH_4_/kg DMI). DMI in our study was 2.4 and 2.5% BW for NEL and AN, respectively, and similarities in methane yield could be due to the similarity in intakes between the two breed compositions.

Larger and fast-growing cattle will generally eat more and produce more enteric methane than smaller, slower-growing cattle under the same feeding regimen [40]. However, as shown in this trial, these animal makeup for higher daily CH_4_ production with improved performance.

In the grazing period, no difference in BW was observed. As the ADG of the AN animals was higher than NEL in the grazing and feedlot period (Table 4) the difference between the BW of the two breed compositions was higher in the feedlot. It was observed that there was no difference for BW during the grazing season, but significant differences for CH_4_ intensity (g CH_4_/kg BW) were detected with AN emitting more. However, methane intensity was similar between the breed compositions in the feedlot even with differences in BW (*P*<0.01) for the animals in this finishing stage.

Regarding CH_4_ per unit of ADG, no difference was observed between the two breed compositions in pasture (*P* = 0.07). In contrast, in feedlot the CH_4_/ADG or CH_4_/carcass ADG was significantly lower (*P*<0.01) in AN than NEL animals.

The AN animals were more efficient and obtained lower CH_4_ per ADG compare to NEL in feedlot. Previous studies did not support the hypothesis that an increase in feed efficiency decreases CH_4_ production [41, 42]. However, [43] showed that more efficient animals produce less enteric CH_4_ production than less efficient animals, especially when these animals are fed a high concentrate diet, which agrees with our results.

Previous research has focused on the use of feedlots as a strategy to reduce CH_4_ emissions per kg of meat produced compared with grazing system. However, the majority of studies evaluated the continuous grazing management system and assumed steady-state soil carbon (C) to model the grass-finishing environmental impact [7]. Additionally, in these studies the ADG is generally below what can be achieved in well managed intensive pasture systems and because of this a substantial reduction in net GHG emissions may occur in pasture systems even when requiring double the land of feedlot systems, as a consequence of increased animal performance and sequestration. Important caveats to this hypothesis include potential soil carbon saturation and the permanence of the carbon sequestered. For instance, [44] estimated a 20-year window of improved soil carbon sequestration before reaching a dynamic equilibrium in the soil. However others have indicated periods of time from 90 to 300 years before reaching saturation [6, 45].

Concerning total methane production (adding the methane emitted in both grazing and feedlot), it was observed that the CH_4_ production was higher for AN animals compared to NEL (43.84 kg versus 39.55 kg). However, methane production per kg of carcass was 0.124 versus 0.139 for AN and NEL, respectively. These results suggest that the methane production of crossbred animals is compensated by better performance, resulting in lower CH_4_ per kg of meat produced, when this intensive production system is used in tropical climate conditions. Identifying efficient cattle breeds and adopting appropriate production systems is an important challenge for meat production worldwide with the growing concern about beef productions impact on the environment [46].

In addition to reducing enteric CH_4_ emissions/kg of meat produced, another advantage of intensification is associated with the reduction of the land area required to produce the same amount of product. This has the potential to reduce the degraded area and, in addition, contribute to the non-opening of new areas and mitigate future deforestation [47]. Although, the main disadvantage of the intensification is the need for substantial initial investment and working capital. The higher production of meat (kg produced/area) in the intensive tropical pasture system resulted in reduction in the unit cost and increase in the rate of return investment, despite the greater need for investment and cost per animal, when compared to the extensive systems [48].

## Conclusions and implications

The present study proposed to compare the GHG mitigation potential of two breed compositions in established Brazilian intensive beef cattle production system. Our data shows that CH_4_ intensity and CH_4_ emission per ADG might be altered depending of breed and diet composition, although breed composition did not influence the CH_4_ yield. In grazing, NEL presented less CH_4_ intensity, but AN animals in feedlot contributes to the reduction of methane per ADG. Overall, the AN animals were more efficient and had greater weight gain compared to Nellore, resulting in lower methane per kg of meat produced over the whole experimental period.

The data generated could contribute to the development of methane mitigation policies, assuming standard systems that combines pasture use in the rearing phase and grain-based diet for finishing the animals. The integrated systems could enable high gains per unit of land, and feedlot finishing contributes to increased productivity of the whole system.

Therefore, associating these two systems for beef cattle breeding in a tropical climate conditions with extensive pasture areas seems to be in line with new GHG reduction policy.

## Supporting information

S1 Fig Climate data for the experimental period from October 2015 to November 2017, measured at the Embrapa Maize and Sorghum Research Centre meteorological station, Sete Lagoas, MG, Brazil. (PDF)

S1 Table Dataset used for statistical comparison of two breed composition. (XLSX)
