# Supplementary figures and images for: Could the breed composition improve performance and change the enteric methane emissions from beef cattle in a tropical intensive production system?
Source: PLoS One. 2019 Jul 26;14(7):e0220247. doi: 10.1371/journal.pone.0220247 (PMC6660127; doi:10.1371/journal.pone.0220247)

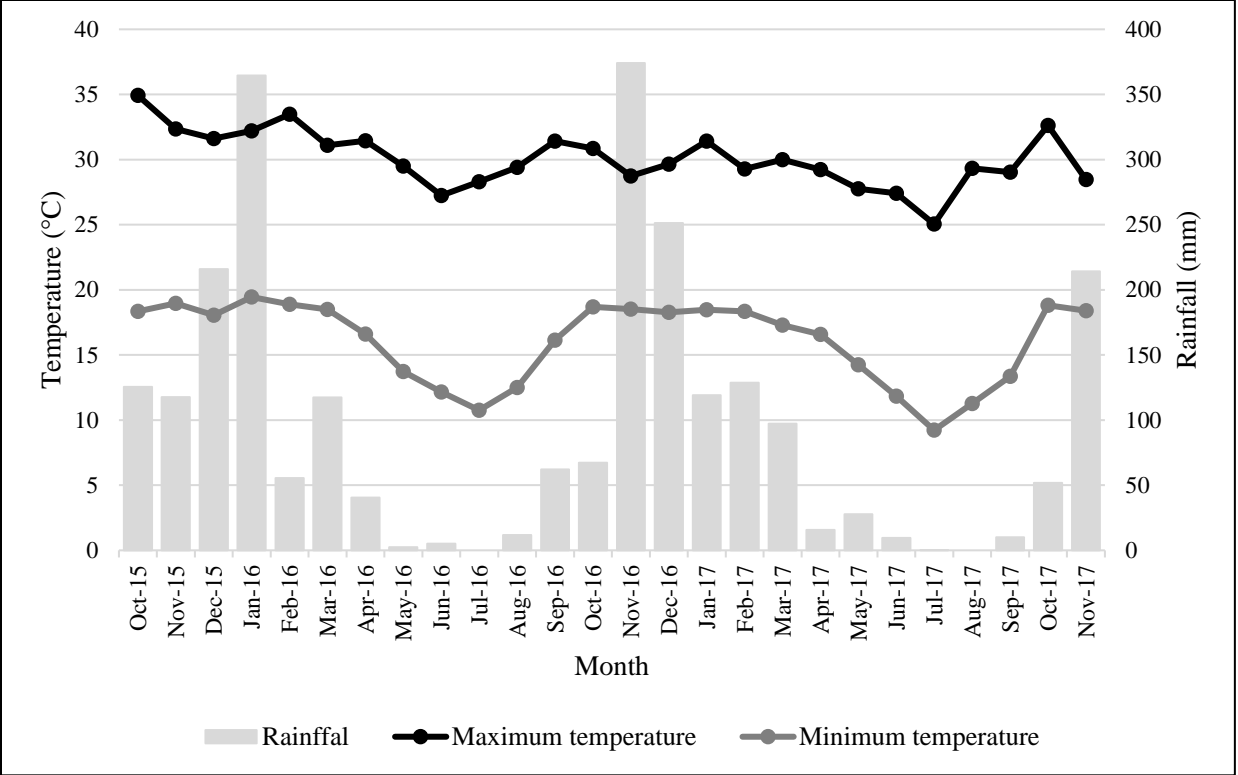

Supplement: S1 Fig — (PDF) [file pone.0220247.s001.pdf]
